# Nanomaterials-Based Sensors for Respiratory Viral Detection: A Review

**DOI:** 10.1109/JSEN.2021.3085084

**Published:** 2021-05-31

**Authors:** Gowhar A. Naikoo, Tasbiha Awan, Israr Ul Hassan, Hiba Salim, Fareeha Arshad, Waqar Ahmed, Abdullah M. Asiri, Ahsanulhaq Qurashi

**Affiliations:** Department of Mathematics and SciencesCollege of Arts and Applied SciencesDhofar University 108727 Salalah PC 211 Oman; College of EngineeringDhofar University 108727 Salalah 211 Oman; Department of BiochemistryAligarh Muslim University 30037 Uttar Pradesh 202002 India; School of Mathematics and Physics, College of ScienceUniversity of Lincoln 4547 Lincoln LN6 7TS U.K.; Department of ChemistryFaculty of ScienceKing Abdulaziz University 37848 Jeddah PC 21589 Saudi Arabia; Department of ChemistryKhalifa University Abu Dhabi PC 127788 United Arab Emirates

**Keywords:** Nanomaterials, respiratory viral detection, SARS-CoV-2, types of sensors

## Abstract

Contagious diseases are the principal cause of mortality, particularly respiratory viruses, a real menace for public health and economic development worldwide. Therefore, timely diagnosis and treatments are the only life-saving strategy to overcome any epidemic and particularly the ongoing prevailing pandemic COVID-19 caused by SARS-CoV-2. A rapid identification, point of care, portable, highly sensitive, stable, and inexpensive device is needed which is exceptionally satisfied by sensor technology. Consequently, the researchers have directed their attention to employing sensors targeting multiple analyses of pathogenic detections across the world. Nanostructured materials (nanoparticles, nanowires, nanobundles, etc.), owing to their unique characteristics such as large surface-to-volume ratio and nanoscale interactions, are widely employed to fabricate facile sensors to meet all the immediate emerging challenges and threats. This review is anticipated to foster researchers in developing advanced nanomaterials-based sensors for the increasing number of COVID-19 cases across the globe. The mechanism of respiratory viral detection by nanomaterials-based sensors has been reported. Moreover, the advantages, disadvantages, and their comparison with conventional sensors are summarized. Furthermore, we have highlighted the challenges and future potential of these sensors for achieving efficient and rapid detection.

## I. Introduction

**Fig. 1.**
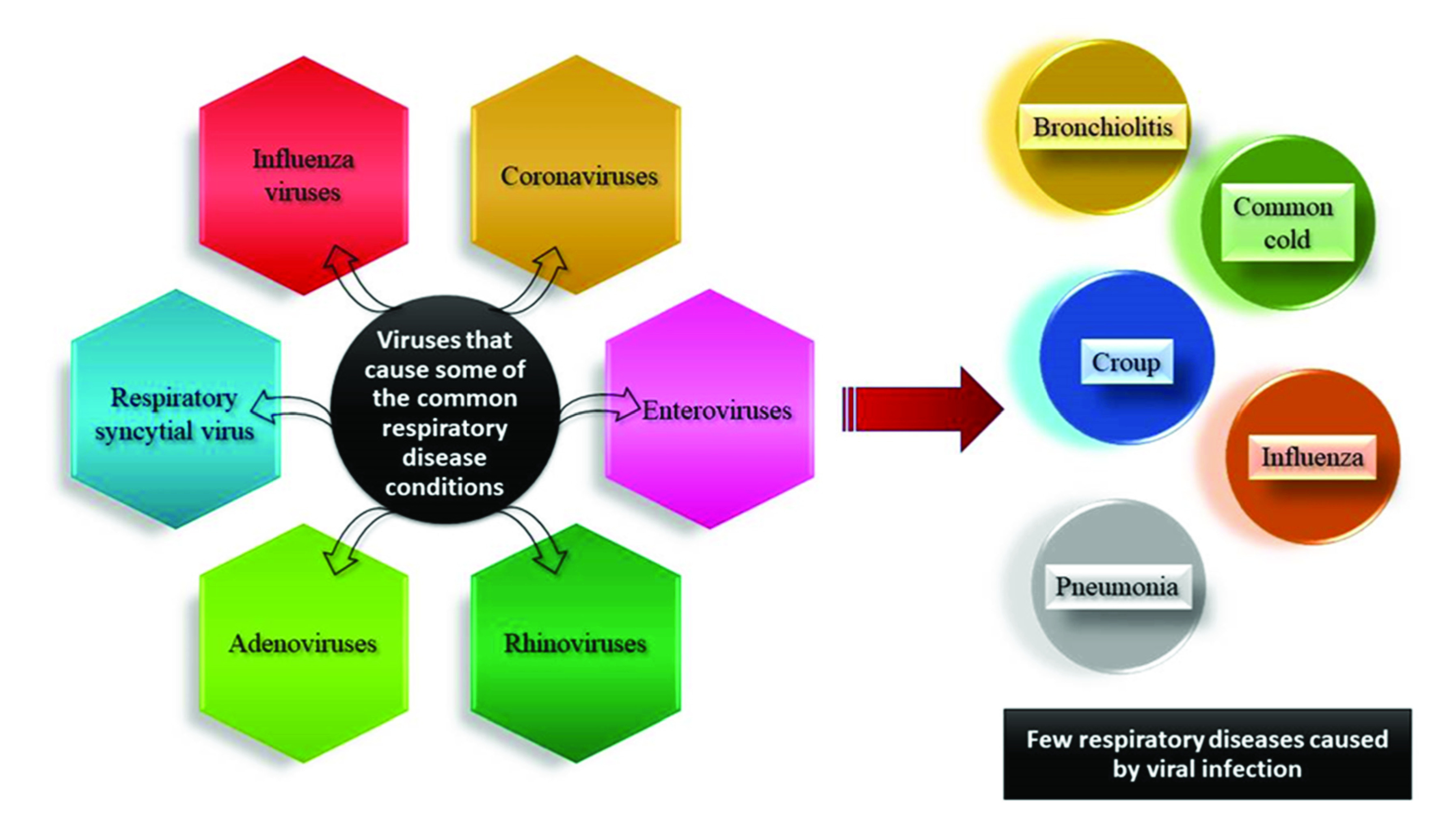
Some common respiratory diseases caused by respiratory viruses.

Viruses are a severe threat to living things on earth, so timely detection for clinical point-of-care purposes [1] is significant in saving someone’s life, as a slight delay can be very detrimental. The respiratory mucosa is the most susceptible site for respiratory viruses, well recognized as influenza virus and coronavirus or severe acute respiratory syndrome (SARS) and some others [2]–[4], typically causing respiratory viral infections that are transmitted through direct contact or air (**Fig. 1**) [5]. The most prominent symptoms of infected individuals are fever, loss of smell, dry cough, fatigue, and sputum production leading to acute respiratory morbidities. Viral infections can be transmitted via contaminated food, water, and bodily fluids, endangering both the lives of humans’ and animals’ worldwide [6], [7]. From the upper respiratory tract, the respiratory pathogens progress down the lower respiratory tract, causing severe pneumonia and ultimately leading to the deaths of around 3 million individuals every year [8]. However, it seems like any ordinary mild cold, but in the past 20 years, we have witnessed severe outbreaks from these acute respiratory viruses to the points of epidemics [9]–[13]. At the start of December 2019, the outbreak of the novel, highly lethal COVID-19 was first recognized in Wuhan, Hubei province in China [14]. Due to the rapid expansion of SARS-CoV-2 worldwide, the World Health Organization (WHO) declared the outbreak as a pandemic on 11^th^ March 2020 [15]–[17]. Infections with SARS-CoV-2 are now widespread, and as of 23^rd^ March 2021, 123,216,178 infected cases, with 2,714,517 deaths, have been confirmed by WHO. As of 20^th^ March 2021, a total of 397,950,709 vaccine doses have been administered. The most vulnerable to this disease are individuals with weak immunity and comorbidities or those already suffering from other underlying illnesses such as heart disease, diabetes, etc. Safety measures such as face masks, sanitizers, and other immunity enhancers are strongly recommended [18].

Viral infections can be detected by various lab-based conventional techniques encircling virus cultures and serological tests followed by Polymerase Chain Reaction (PCR) [19], Reverse Transcription-Polymerase Chain Reaction (RT–PCR) [20], western blotting and antibody detection, Fluorescent Antibody Tests (FAT), antigen or antibody detection and hemagglutination assay and gene sequencing [21], [22], isothermal amplification techniques [23] and immunochromatography (IC) [24]. These classical detecting techniques, especially PCR, have helped physicians identify the causative agents. However, sample preparation, high cost, time-consuming, labor-intensive, and less accurate detections have limited their reliability [25]. Test kits for virus detection (Covid-19) are based on RNAs following PCR. These procedures rely on the interaction between the complementary detection ligands or strands in the equipment and their surfaces [26]. As mentioned earlier, these test kits are unreliable and require lengthy processing times to provide inaccurate results [16]. Therefore, it is essential to develop a more precise, fast, simple, portable, and sophisticated testing platforms to diagnose threatening respiratory viruses [7], [27]–[29].

Nanomaterial-based techniques have emerged as promising candidates proving significant improvement in detection devices called nano-sensors [26], [30], and currently, various sensor technologies are explored to detect viruses [30]–[33]. The sensors operated by the combination of interacting recognition elements with the sensing system, thus identifying the target with great accuracy and sensitivity [34]. The exceptional conductivity and photoelectrochemical properties, portability, and simplicity of nanomaterials are exploited for the enhanced efficiency and sensitivity of nano-sensors to diagnose respiratory viruses [35]–[37]. The prominent challenge researchers face developing ultra-sensitive, rapid, and stable nano-sensors is by enhancing the nanomaterials’ surface area, thus providing significant surface interactions to analytes and sensors [38] and exploiting it as indicators in sensors [39]. The nano-sensors can detect bacteria and viruses at low concentrations, thus making them the best choice for diagnostic purposes [40].

The purpose of this review is to address the different types of nanomaterial-based sensors explored recently, encompassing the latest information summarized in separate tables for respiratory virus diagnosis published in the past five years. Furthermore, this review paper extensively discusses the physical structure and principle of virus detection, antibody-antigen interactions, sensation, and mechanism of nanomaterial-based sensors, followed by their advantages and limitations for respiratory viral detections. A comparison of nanomaterial-based sensors with conventional classical techniques is also mentioned. Finally, future challenges and perspectives of nanomaterial-based viral sensors and a brief conclusion are presented to summarize the full review. It is always helpful to overview already accessible literature to explore and develop new and more advanced techniques for the diagnostics and treatments of new viruses. Consequently, it is anticipated that this review regarding respiratory viruses will illuminate the researchers with the latest knowledge that will help develop unique, sensitive, and highly stable nano-sensors for fast diagnosis of COVID-19 patients.

## II. Types of Sensors for Respiratory Viral Detection

To prevent and control any future pandemics due to respiratory viruses, we must have quick and reliable detection methods. This can be achieved using inexpensive, sensitive sensors that give rapid and accurate results. Such sensors will help us better control the diagnosis and treatment of the existing and future respiratory virus infections. Any sensor platform is influenced by three major factors: (1) the target analyte which has to be identified, (2) the process of identification used, and (3) the amplification of those signals produced during the identification process so that they can quickly be recorded. Hence, ideal sensors for detection of respiratory viruses must have the ability to give reproducible results, is autonomous in use, give immediate results, have superior sensitivity and selectivity, can detect multiple analytes simultaneously, possess many sensing modes, is inexpensive, have a long life, is user-friendly, and can be disposed of easily without any harm to the environment. Four such respiratory viral detection sensors have been utilized in recent years. These include nanomaterial-based sensors, electrochemical sensors, optical sensors, and piezoelectric sensors.

### A. Nanomaterials-Based Sensors

**Fig. 2.**
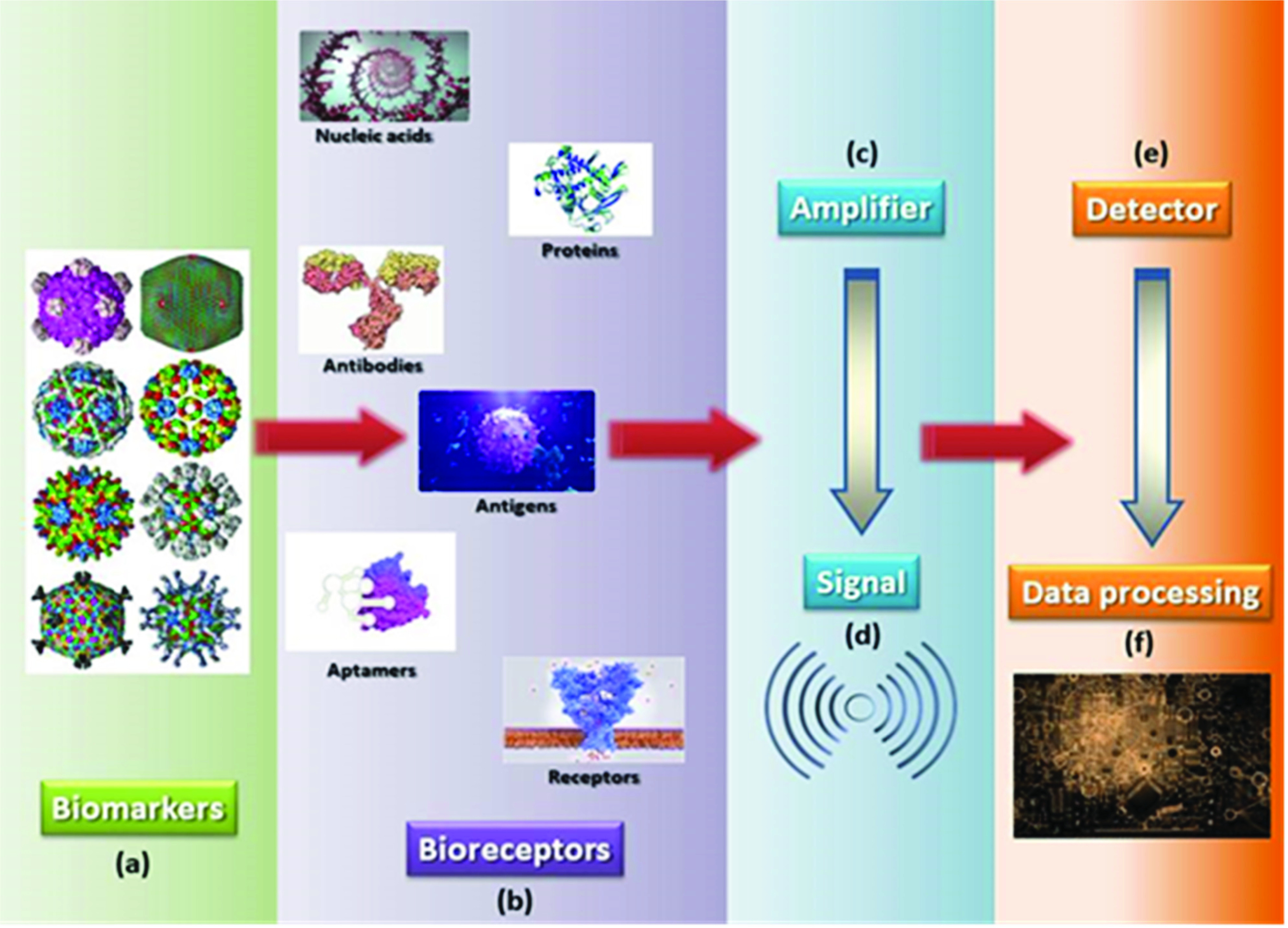
Schematic diagram of nanomaterial-based sensor for respiratory virus detection consisting of (a) biomarkers, which are usually virus particles, (b) receptors, which are either composed of nucleic acids, proteins, antibodies, antigens, or aptamers, (c) nanomaterial that is attached to the electrode of the sensor, (d) the transducer that detects the signals produced by the interaction between the analyte and the bioreceptor, (e) the electronic system that translates the signal produced in the transducer into a readable electrical signal.

Rapid viral diagnostics is essential to overcome the current pandemic and prevent future ones. Functionalization of nanomaterials using nucleic acids or proteins like antibodies is the standard method used for viral detection. These detections are carried out using colorimetric assays, thermal photo platforms, and antigen-binding assays, among others. The commonly available testing kits for viral infections operate on enzyme-linked immunosorbent assays or PCRs [41]. These testing kits have been shown to have false-negative results, low sensitivity, and usually take long durations to give results [16]. Nanomaterials, on the contrary, are known to lower these drawbacks. Having a high surface-to-volume ratio allows nanoparticles to have better surface interactions between the sensor and the biomolecule to be analyzed. This makes nanomaterial-based sensors a better candidate for rapid and dependable detection of viral particles [26]. Many nanomaterials with transducing functions have been studied in recent decades, and several matrices for different sensors have been developed [42]. Nanomaterials like graphene, quantum dots, gold and silver nanoparticles have been explored to create sensors to detect viral particles. There have been several nanosensors developed and reported in the literature over the last decade – each of them employing a unique nanomaterial to increase its sensitivity and selectivity to give a rapid response rate during viral detection (**Fig. 2**).

Apart from these, noble metal nanomaterials have also been explored to detect viral particles using sensors. These nanomaterials have also been shown to increase the specificity and sensitivity of the sensing systems. Hence, the fabrication of sensors with nanomaterials has significantly improved the sensing capabilities of these devices [43]. Nanoscale devices exhibit efficient functionality with unique effects compared to standard devices and play a central role in eliminating errors in viral detection methods. Nano-sensors can be used on a large scale for disease diagnosis and biomolecule detection due to bio-elements and transducer interactions. Various nanomaterials, such as nanorods, nanotubes, nanowires, thin films, and nanoparticles, are being used in biomedical applications because of their mechanical and electrical characteristics [44]. Many recent researches on various nanomaterial-based sensors have been discussed in Table I.

**TABLE I:** Electrode Materials for the Respiratory Viral Detection

Sensor method	Electrode materials	Virus	Recognition element	Linear range	LOD	Time	References
EIS	shellac-derived TrGO/ITO/glass	Influenza virus H1N1	Anti-Influenza A Ab	–	PBS: 26.04 PFU/mL Saliva: 33.11 PFU/mL	–	[56]
EIS	Au- paper electrode	H1N1 Antigen	Anti-influenza A HA Ab	–	< 5 PFU mL^−1^	~6 min	[57]
EIS CV	Cysteine modified Au-screen electrode	H1N1	HA gene specific ssDNA probe	0.1 ng – 400 ng/ $6~\mu \text{L}$	0.004 ng in $6~\mu \text{L}$	30 min	[58]
EIS	nanocrystalline boron-doped diamond	Influenza Virus M1 protein H1N1, H3N2	Polyclonal aM1 Ab	–	$5\times 10 ^{-14}$ g/mL	6 min	[59]
EIS	Nano-scale BDD sensor	Influenza A M1 protein	anti-M1Ab,	1 to 100 fgmL^−1^	1 fg/mL	5 min	[60]
EIS	Au electrode	inactivated intact H1N1virus	DNA aptamers	–	0.3ng/ $\mu \text{L}$	–	[61]
EIS	AuSPE/Gr/Au hybrid nanocomposite	Influenza A H9N2	Feutin A	10^−8^ U/mL-10^−1^ U/mL	10^−8^U/mL	–	[62]
CV	Fe_3_O_4_ MNPs	AIV H5N1	Specific Anti-H5N1 Ab	0.0025 - 0.16 HAU	0.0022 HAU in $6~\mu \text{L}$	–	[63]
CV	AuNPs	H5N1	DNA probe	1pM - 100 nM	1 pM	–	[64]
CV	Dual electrode of tungsten rods modified with 3DNRE porous silica film	AIV	anti-AIV NP aptamer	2 to 12 nM	1.13 nM	–	[65]
DPV	Hydrophobic SiNPs on paper	Influenza H1N1 antigen, Ab	Immobilized antibodies	10 to 10^4^ PFU mL^−1^	113 PFU mL^−1^	30 min	[66]
DPV	modified ITO/glass electrodes	H1N1 mini-HA protein	ssDNA aptamer	10^1^ PFU mL^−1^ – 10^4^ PFU mL^−1^	3.7 PFU mL^−1^	–	[67]
CV DPV	FTO/AuNPs sensor	SARS-CoV-2	immobilized with nCovid-19Ab	1 fM – $1~\mu \text{M}$	10 fM at nCovid-19Ag	10 - 30 s	[68]
CA	AuNPs	influenza virus A/H9N2	anti-Matrix protein 2 (M2) Ab and Feutin A	8 – 128 HAU	8 HAU	160 s	[69]
CA	RGO nanosheets	Human Influenza A H1N1	monoclonal antibodies	1 – 10^4^ PFU mL^−1^	0.5 PFU mL^−1^,	–	[70]
BERA	Membrane-Engineered Vero Cells (Vero/Anti-S1)	SARS-CoV-2 S1 Protein Antigen	spike S1 Ab	10 fg – $1~\mu \text{g}$/mL	1 fg/mL	3 min	[71]
Fluoroimmunoassay	gCNQDs/MP-MoO_3_QDs	influenza virus A (H3N2) RNA	Ab-(H3N2)	45– 25,000 PFU/mL	45 PFU/mL	–	[72]
Fluoroimmunoassay	S-gCNQDs/Ag_2_S nanocrystals	influenza A virus (H1N1) (H3N2)	monoclonal Ab	10 fg/mL– 1ng/mL 10^2^ – $5\times 10^{4}$ PFU/mL	5.5 fg/mL 100 PFU/mL	~ 15 min	[73]
FET	Graphene Sheet	spike protein in SARS-CoV-2	SARS-CoV-2 spike Ab	–	cultured medium: $1.6\times 10 ^{1}$ pfu/mL clinical samples: $2.42\times 10 ^{2}$ copies/mL	–	[53]
FET	rGO transistor	H5N1	Immobilized DNA probe	10 pM – 100 nM	5 pM	1 h	[74]
FET	Cu/Au/SiO_2_	AIV H5N1	Immobilized DNA aptamer	10 pM – 10 nM	5.9 pM	–	[75]
Gr-FET	Gr synthesized on single crystal Cu^+3^	coronavirus	CSAb/ACE2	< 0.1 pM	0.2 pM	2 min	[76]
Amperometry	3,4- EDOT PSS thin film	influenza A virus	HA protein	–	0.025 HAU	–	[77]
Amperometry	Co-TNTs	spike glycoprotein SARS-CoV-2	anti-His monoclonal Ab	14 – 1400 nM	~0.7 nM	~30 s	[78]
SWV	AuNPs on CE	MERS-CoV HCoV	Anti-recombinant spike protein Ab	MERS-CoV: 0.001 – 100 ng.mL^−1^ HCoV: 0.01– 10,000 ng.mL^−1^	MERS-CoV 1.0 pg/mL HCoV 0.4 pg/mL	20 min	[50]
OSWV	Au-electrodes	H5N1	immobilization of ss-amino-DNA probe	$3.0\times 103$ – $3.0\times 105$	3 copies/ $\mu \text{L}$	–	[79]
DPV EIS	AuNPs	Corona virus viral RNA/c-DNA	immobilized capture probe	–	–	–	[80]
Optical Silica Inverse Opal	SiO_2_-IO nanostructures	HA protein in influenza A (H1N1)	(H1N1) virus capture Ab (HA-1)	103–105 PFU	–	–	[86]
LSPR; colorimetric assay	AuNPs	MERS-CoV	thiol-modified probes	–	1 pmol/ $\mu \text{L}$	–	[87]
IM-SPR	Bimetallic silver–gold	HA protein in H7N9 virus	monoclonal Ab	$2.3\times 102$ to $2.3\times 105$ copies/mL	402 copies/mL	< 10 min	[88]
SPR	Au	influenza A (H5N1)	H5N1 virus protein	–	193.3 ng mL^−1^	–	[33]
LSPR/FL-LSPR	AuNP	influenza virus H3N2	–	–	0.4 pg/mL 10 PFU/mL	~5 min	[89]
LSPR	hAuSN immobilized on ITO	AIV H5N1	DNA 3 way-Junction (3WJ)	–	1 pM	–	[90]
fluorescence assay	DNA triplex assembly	avian influenza A (H7N9)	berberine	0.2–100 nM	0.14 nM	–	[91]
colorimetric assay	AuNPs	SARS-CoV-2	antisense oligonucleotides	0.2–3 ng/ $\mu \text{L}$	0.18 ng/ $\mu \text{L}$	10 min	[92]
lateral flow immune assay	AuNP colloids	SARS-Cov-2	COVID-19 recombinant antigen	–	–	15 min	[93]
CRISPR-based assay	CRISPR-based fluorescent sensor	SARS-CoV-2	Cas12a/gRNA complex	–	2 copies per sample	~50 min	[94]
PPT effect/LSPR	gold nanoislands (AuNIs)	SARS-CoV-2	complementary DNA receptors	–	0.22 pM	–	[95]
colorimetric assay	silver nanoparticles (AgNPs)	MERS-CoV	pyrrolidinyl peptide nucleic acid (acpcPNA)	–	1.53 nM	–	[83]
P-FAB	AuNPs based on the U-bent optical fiber sensor system	Target Nucleocapsid protein N-protein	anti-N protein monoclonal antibodies	–	10–18 M	15 min	[96]
EWA LSPR	AuNPs or polyaniline-coated optical fibers	specific surface proteins SARS-CoV-2	antibodies for unique S1 subunit of the S protein	–	37 pM	–	[97]
Piezoelectric – SPM	Lead zirconate titanate piezoelectric disk	H5N3	$3^{\prime}$ SL-PAA polymer/H5N3 surface glycoprotein	10.05 – 10.07 vp/mL	10.05 vp/mL	–	[100]
Piezoelectric immunosensor	SAM of mercaptoundecanoic acid (MUA)	Aerosolized influenza A (H3N2) Virions	Polyclonal anti-influenza A (H3N2) antibodies	0.02 - 3 HAU	29.6 ng/mL	–	[103]
Piezoelectric Immunosensor	Horse polyclonal antibody against SARS-CoV attached to PZ crystal surface	SARS-associated coronavirus (SARS-CoV)	SARS antigen	0.6 - 4 $\mu \text{g}$/mL	–	–	[104]
Quartz crystal microbalance (QCM) based piezoelectric sensor	Gold surface of the crystal	African swine fever virus	Protein p12	4.6 - 300 $\mu \text{g}$/mL	–	–	[105]

EIS — Electrochemical Impedance spectroscopy, SiNPs — Silica Nanoparticles, CV — Cyclic Voltammetry, CA — Chronoamperomtery, DPV — Differential Pulse Voltammetry, FET — Field-Effect Transistor, Gr-FET — Graphene Field-Effect Transistor, BERA — Bioelectric Recognition Assay, OSWV — Osteryoung Square Wave Voltammetry, SWV — Squarewave Voltammetry, PBS — Phosphate-buffered Saline, S-gCNQDs — Sulfur-doped Graphitic Carbon Nitride Quantum Dots, MNPs — Magnetic Nanoparticles, BDD — Boron Doped Diamond, Ab — Antibody, CSAb — Spike S1 Subunit Protein Antibody, Co-TNTs — Cobalt-functionalized TiO2 Nanotubes, aM1 Ab — Anti-M1 Antibodies, NP — NucleoProtein, rGO — Reduced Graphene Oxide, CE — carbon electrode, AIV — Avian Influenza Virus, EDOT — Ethylenedioxythiophene, HA — Hemagglutinin, ss — single stranded, 3DNRE — 3D Nanostructured, ITO — Indium Tin Oxide, nCovid-19Ab — nCovid-19 monoclonal antibody, FTO — Fluorine-doped Tin Oxide electrode, AuNPs — Gold Nanoparticle, TrGO — Thermally-decomposed rGO, MP-MoO3QDs — Plasmonic Molybdenum Trioxide Quantum Dots, gCNQDs — graphitic Carbon Nitride Quantum Dots, AuSPE — Gold Screen Printed Electrode LSPR — Localized Surface Plasmon Resonance, IM-SPR — Intensity-Modulated Surface Plasmon Resonance, FL-LSPR — Induced Immunofluorescence, PBS — Phosphate-buffered Saline, SiO2-IO — SiO2-based Inverse Opal, hAuSN — hollow Au Spike-like Nanoparticle, ITO — Indium-Tin-Oxide, AuNIs — Gold Nanoislands, PPT — Plasmonic Photothermal, P-FAB — Plasmoni Fiber Optic Sensor, EWA — Evanescent Wave Absorbance

### B. Electrochemical Sensors

Electrochemical sensors record the changes in uniformity of the charge on the surface of transducers. These changes are recorded depending on the impedimetric [45], [46], potentiometric, or amperometric [47], [48] principles of transducers [49]. According to most published literature present, these sensors are most commonly used for the detection of influenza viruses. Another study to develop a nano-immunosensor to detect MERS-CoV was done by Layqah and coworkers [50]. The biomarker targeted using this sensor was the spike protein S1, most commonly targeted by antibodies [51]. The sensor was based on the relation between the free virus present in the given sample and the spike protein S1. The sensor used carbon electrodes that were functionalized using AuNPs that increased the electrochemical function of the sensor and provided a wider area for the biomarker detection using the sensor. Moreover, the sensor was shown to have a better transfer rate. This immunosensor provided an excellent linear range of detection of 0.001 and 100 ng/mL for the MERS virus and displayed an enhanced sensitivity of 0.4 pg/mL. Based on AuNP particles, this sensor showed a very high sensitivity compared to the conventional ELISA method of virus detection [52]. This sensor stood out by its ability to detect two different viruses simultaneously – the MERS-CoV and HCoV on the same electrode surface and was electrochemically active for long durations.

In another study, Seo and colleagues developed a graphene-based sensor that could detect SARS-CoV-2 viral particles from nasopharyngeal swabs of coronavirus patients. In this FET-based sensor, a graphene sheet was used that was attached to a SiO_2_/Si substrate. This was further functionalized with the SARS-CoV-2 spike antibody. The viral particles could be detected using this sensor even at very low concentrations of 1 fg/mL [53]. The detection limit was found to be 100 fg/mL. Furthermore, this sensor was found to be highly selective to SARS-CoV-2 alone and showed no response to MERS-CoV spike proteins.

Electrochemical sensors that use nucleic acids as their target analyte, allow for easy binding of the DNA or RNA on the sensor’s surface. When the specific binding occurs between the two, the electrode’s surface experiences a change – which is then recorded using electrochemical methods. These electrochemical signals produced are a consequence of the transfer of electrons between the probe and electrode. Aptamers, hairpin DNA, locked and peptide nucleic acids are the most widely used probes in electrochemical sensors [54]. Of these, aptamers have gained more attention, owing to their high selectivity for the target analytes. They target proteins, DNA or RNA, and other chemical molecules, alike. Hence, electrochemical sensors have shown to be very stable, highly specific, and can be easily miniaturized [55]. Table I illustrates the various options of electrochemical sensors for detection of virus particles.

### C. Optical Sensors

Optical sensors are also essential tools in detecting multiple analytes simultaneously. Such sensors analyze the changes in optical properties of transducers during the exchange between the target molecule and the recognition element [81], [82]. The application of optical sensors to detect virus particles was seen in the work put forward by Teengam and colleagues [83]. The authors developed a unique optical sensor that could detect MERS particles. The experiment results could be seen by the naked eye and did not use any complex experimental gadgets. It was a paper-based colorimetric sensor that noted the response of aggregation or de-aggregation of silver nanoparticles with viral DNA molecules. This reaction occurred in the presence of pyrrolidinyl peptide nucleic acid. Another good instance of optic sensor for respiratory virus detection was the work carried out by Ostroff and colleagues. They are the only group that put forward a sensor to detect human rhinovirus using optical sensors [84]. They used an optically coated silicon surface functionalized by virus antibodies. The sensor was shown to give the results under 30 minutes and showed a good sensitivity range. Hence, these sensors enhance sensitivity and increased efficiency. Optical-based techniques like Surface Plasmon Resonance (SPR) [46], Surface-Enhanced Raman Scattering (SERS) [85] are also used to detect virus particles. Table I demonstrates the various optical detection techniques for respiratory viruses (Influenza Virus, SARS-CoV-2).

### D. Piezoelectric Sensors

Piezoelectric sensors function in the presence of an applied alternating electric field. The transducers in such sensors resonate under such conditions. They measure the difference in the resonating frequency, which occurs because of crystal mass and the attached target analyte. When the target analyte binds to the sensor’s surface, a change in the mass occurs, which is recorded. These sensors are widely used to detect various target molecules within biological systems [55], [98], [99]. The use of this sensor for the detection of the H5N3 virus is seen in the work done by Erofeev *et al.* [100]. The duo developed a piezoelectric sensor using a lead zirconate titanate piezoelectric disk and successfully performed a label-free detection of the influenza virus. In another study, it was observed that a piezoelectric sensor coated with aptamer could detect the SCV helicase protein derived from the SARS CoV [101]. The sensor could perform the detection in a minute and showed a LOD of 3.5 ng/mL.

Piezoelectric nanosensors also monitor the changes in viscoelasticity by noting the frequency, which affects the quartz crystal resonator [102]. During the sensing activity by such sensors, isolation equipments are used to avoid any hindrance caused by the external environment. Table I discusses the comparison of different piezoelectric detection techniques for respiratory viruses.

## III. Physical Structure of the Sensors and the Virus Detection Principles

**Fig. 3.**
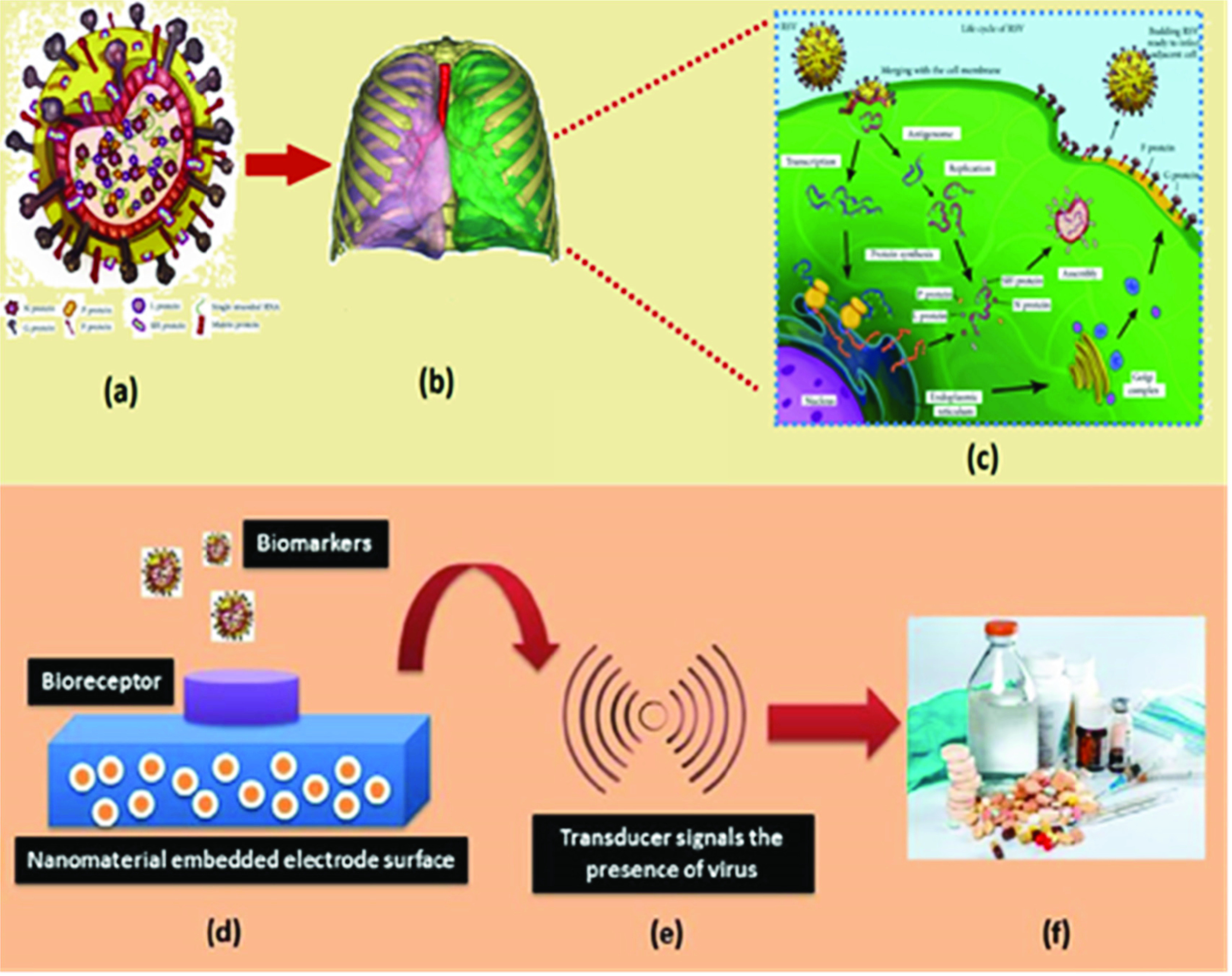
(a) The structure of Respiratory Syncytial Virus (RSV). Reproduced from ref [110] Copyright © 2013 Swapnil Subhash Bawage *et al.* (b) After the onset of RSV attack, the lungs and the respiratory tracts gets severely infected. (c) The life cycle of RSV within the lung system. Reproduced from ref [110] Copyright © 2013 Swapnil Subhash Bawage *et al.* (d) Materials science, like the sensor technology has contributed towards easy identification of the virus particles. (e) When an interaction occurs between the analyte (virus particle) and the respective bioreceptor, the transducer undergoes changes and signals the presence of virus infection (f) When the presence of infection is confirmed by the sensor, vaccine and treatment development and delivery are made available to the infected individual.

A sensor that can detect virus functions like any other analytical device and possesses three significant parts: a bioreceptor that senses the analyte, a transducer, and the detector that gives the digital result. The interaction between the target biomolecule and the bioreceptor results in either a physical or a chemical reaction between them [106], [107]. The transducer then converts the changes in molecules into a detectable signal that can be quantified or measured with the help of a digital detector [108]. The transduction process can be based on either the following sensors: electrochemical, immuno, thermal, optical, magnetic, piezoelectric. Each of these types determines the principles based on which the sensor functions [109]. The use of nanomaterials increases the overall sensitivity and selectivity of the sensor by enhancing the overall fabrication quality of the sensor. This can be better understood as depicted in **Fig. 3**, which discusses the attack by a respiratory virus (like the respiratory syncytial virus), its life cycle, physical structure of a nanomaterial-based sensor and its mechanism of sensing.

## IV. Antibody Orientation and Bonding Approach

**Fig. 4.**
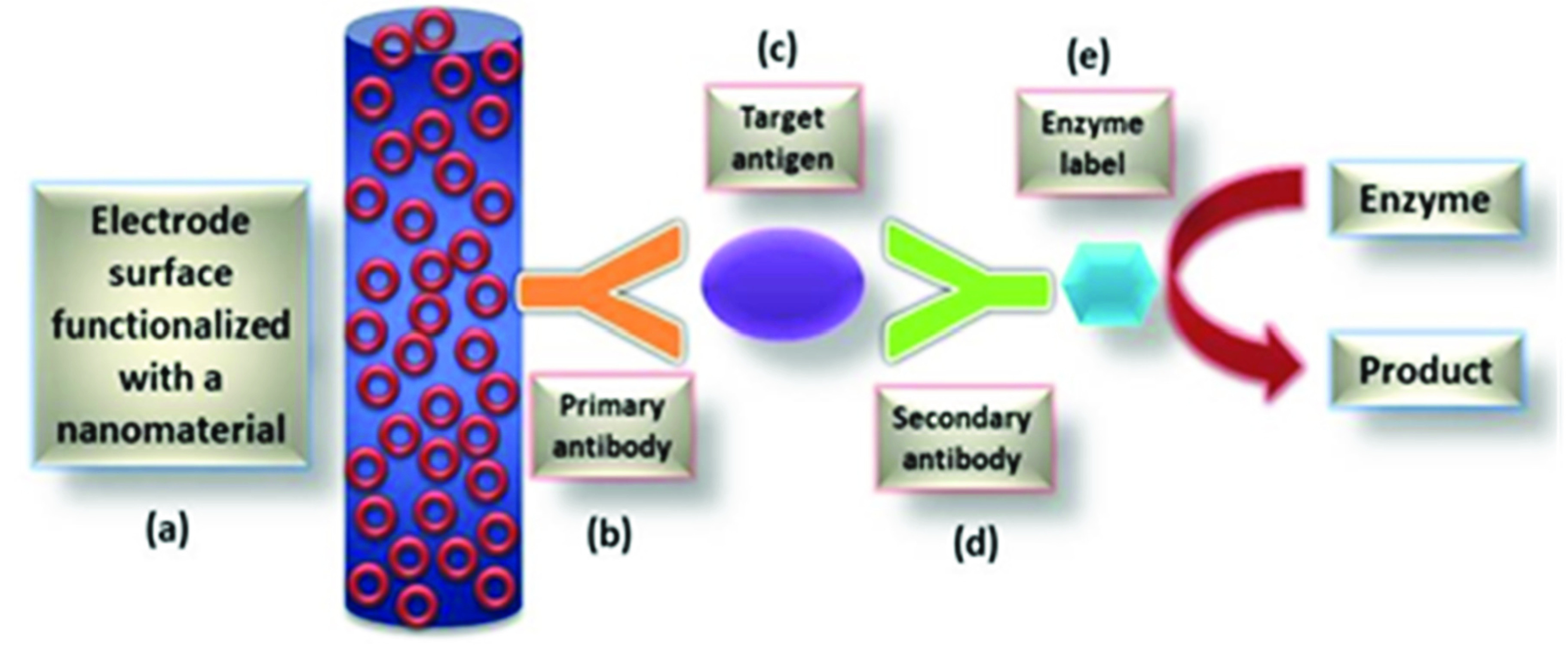
The essential components of immunosensors with enzyme labels. An immunosensor is made up of the following elements: (a) nanomaterial functionalized electrode (b) the primary antibody, which at one end is attached to the electrode surface and the other binds to the target antigen, (c) the target antigen is linked to the secondary antibody, (d) the secondary antibody at the other end is labeled with an appropriate enzyme, (e) the enzyme undergoes changes that are recorded on the transducer.

The nanomaterial-based immunosensors are used for the detection of viral particles. The function of these sensors is affected by one or more of the following conditions: the affinity of antigens, the orientation and accessibility of the binding sites upon immobilization, and the total number of binding sites available on the surface of the sensor. The strategies based on which the immobilization occurs may differ, giving varying results and efficiencies. These also affect the orientation to which the attached antibodies adapt. For instance, when antibodies take a ‘flat-on’ orientation then the adsorption method is used for antibody immobilization [111]. This antibody orientation can become an obstacle for the antigens to approach antibody binding sites (**Fig. 4**). This, therefore, causes a lowering of antigen-binding capacity [112]. However, achieving a 100% specific orientation is not possible. This can be attributed to site-specific changes when antigen binds to the sensor and results in attachment of other reactive groups. Such immobilization strategies are also influenced by the kind of nanomaterial used and should be compatible with the target analytes to avoid the reduction in specific orientations.

**Fig. 5.**
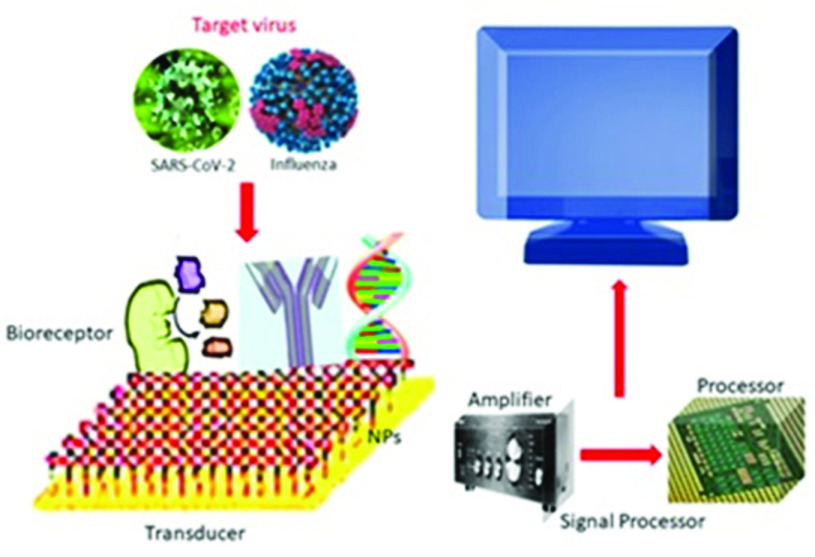
Mechanism of sensing of nanomaterials-based sensors for respiratory viral detection.

## V. Sensing and Measurement Mechanisms

Increasing the conductivity of the sensors influences their sensitivity and limit of detection. Nanomaterials contribute towards aiding the sensing and measurement mechanisms of the sensor. There are many different sensing mechanisms on which sensors operate during virus detection. SPR being an optical technique, has significantly contributed to the processing of immunoassays. Such sensors can simultaneously analyze multiple biomolecules and perform real-time monitoring of various analytes having or devoid of labels [113]. Their sensing happens when a thin layer of metal is layered on a dielectric waveguide, and data is used after reflecting p-polarized light. On the contrary, total internal reflection ellipsometry makes use of the reflecting s-polarized light [114], [115].

There exist other sensing mechanisms like the alternating current electrokinetics capacitative sensing method. This method performs rapid sensing within one step and does not need any washing step [116]. The interfacial capacitance between the electrode and the sample is considered to measure the target analyte amount present on the electrode surface. Based on this data, the detection protocol is developed. Such sensors have been explored in recent years for virus detection [117], [118]. However, these studies have been based on bulk capacitance and are highly influenced by the matrix effect.

## VI. Mechanism of Sensing of Nanomaterials-Based Sensors for Respiratory Viral Detection

During virus detection using nanomaterial-based sensors, three basic steps occur, each linked with explicitly with the previous step. First, the target molecule is recognized by a fixed set(s) of biorecognition components. Interactions between the two occur by either covalent or non-covalent interactions. Second, any chemical change recorded by the transducer of the sensor is transferred to the detector. Finally, the detector displays the results in digital signals on a digital screen [55]. Many transducers are used during the detection, like carbon or gold electrode systems, screen printed electrodes and others. In nanomaterial-based sensors, the transducers use electrodes embedded with nanoparticles or a nanomaterial that increases the specificity with which the bioreceptor interacts with the target biomolecule during the detection process. Also, they enhance the overall sensitivity and reproducibility of the sensor.

Among the kind of sensors used, nanomaterial-based electrochemical sensors have gained wider attention because of their ability to rapidly carry out the sensing mechanism and recognize the target molecule within a very short duration. Such sensors are based on the direct generation of electrical signals within a short period [119]. Furthermore, most electrochemical sensors can perform label-free detection of analytes that permits the development of point of care devices [120].

## VII. Advantages and Disadvantages of Nanomaterials-Based Sensors for Respiratory Viral Detection

As discussed previously, many studies support the superiority of nanomaterial-based sensors over the others due to their unmatched physicochemical properties like the prominent surface to volume ratio, high adsorption, the changes in their quantum aspects and their capacity to react. Hence, a lower quantity of analytes is more than sufficient for their successful analysis. Furthermore, rapid processing of the analyte within a lower budget is possible using such sensors [121]. Even carbon-based nanomaterials like graphene and its derivatives are an excellent choice to develop sensors that can detect respiratory viruses because of their ability to attach to ligands and other nanoparticles [26]. Other nanomaterials like silica nanoparticles are also advantageous in creating sensors to detect viruses because they are biocompatible with other elements in biological conditions. Moreover, nanoporous materials have an even better surface to volume ratio which is gives them an upper hand over the other nanoparticles that are currently available [26].

Though nanomaterial-based sensors have attracted the attention of researchers globally, they still have a few downsides such as low sensitivity as compared to conventional methods like RT-PCR assays. Many electrochemical sensors have also been shown to have lower shelf-life than conventional sensors [76]. Moreover, nanomaterial-based sensors are sensitive to sample matrix effects too [122].

## VIII. Comparison of Nanomaterials-Based Sensors With Conventional Sensors for Respiratory Viral Detection

Apart from the recent nanomaterial-based sensors, traditional methods like cell-culture and colony counting techniques, electron microscopy, immunological assays, PCR, ELISA, nucleic acid-based detection, and others have also been popularly used to detect respiratory viral particles. However, detecting the analyte via these methods requires an additional sample processing step. Samples like nasal swabs are collected and put through sample enrichment processes, only after which the detection assays are carried out. This step is crucial because it increases the efficiency of the sensing process and also reduces the time taken to detect the analyte. Besides being time-consuming, these processes are often meticulous and are prone to errors.

On the contrary, nanomaterial-based sensors allow for direct determination of the analyte without the requirement of any processing steps [123]. The use of conventional sensors is limited because they cannot be used under the banner of ‘point of care techniques’ and fail to produce real-time analyses. An ideal sensor should have: an online sampler that can sample the analyte, a quick system and analyzes the given sample, a completely automatic operation, less processing steps, and elements that enhance the overall sensitivity and specificity of the sensors. This is where nanomaterials play a fundamental role. They can provide high efficiency and improve the overall quality of the sensors. Usage of such nanomaterial-based sensors will reduce the detection time and encourage in real-time analysis.

## IX. Conclusion and Future Prospective

Highly sensitive sensors can be successfully developed using nanomaterials, such that they give rapid results which are highly reproducible. In addition to being highly sensitive, nanomaterial-based FET sensors are also highly selective and can detect the analyte in low concentrations. Nanomaterials like graphene and In2O3 have been incorporated in the open gated areas of FET to detect respiratory viruses [124]. Elements like gold nanoparticles and their nanoislands have also been successfully included in immunosensors to detect viruses like MERS and COVID-19 under pico and femto concentration ranges [95], [125]. Furthermore, using nanomaterials in sensor development allows for their miniaturization and reduces their overall production cost. Affinity sensors can be easily created using multiple nanomaterials within the same sensor. Such sensors usually have many electrodes within the same system, which allows for simultaneous detection. Hence, such nanomaterial-based sensors can be created as small point-of-care devices that hold practical applications, are user-friendly, and do not require trained professionals to detect respiratory viral pathogens.

Moreover, certain aspects of such sensors still need further attention. More work needs to be done to determine the accuracy of the results of nanomaterial-based sensors that can detect actual samples in real-time under natural conditions. Most of the work is usually carried out under laboratory conditions, and hence whether or not they may give similar results even outside the laboratory conditions must be cross-checked. Furthermore, wearable biosensing devices can be created using nanomaterials that offer statistical information like the development of virus within the body, the antibody levels, and compare the respiratory virus infection within the community. This will be particularly useful for uninfected individuals to avoid such areas to prevent further spreading of the disease to neighboring regions. Moreover, such innovative nanomaterial-based sensors can help predict the next pandemic to a large degree. In conclusion, nanomaterial-based sensors can prove to be very promising for the detection of respiratory viral particles. Yet, further work is required to make these user-friendly and provide quick, accurate and early detection of the viruses to prevent any other respiratory virus outbreaks.

## X. Abbreviations

**Table:** 

Abbreviated form	Full form
SARS	Severe acute respiratory syndrome
WHO	World Health Organization
PCR	Polymerase Chain Reaction
RT-PCR	Reverse Transcription-Polymerase Chain Reaction
FAT	Fluorescent Antibody Test
RSV	Respiratory Syncytial Virus
